# Clarithromycin Solid Lipid Nanoparticles for Topical Ocular Therapy: Optimization, Evaluation, and In Vivo Studies

**DOI:** 10.3390/pharmaceutics13040523

**Published:** 2021-04-09

**Authors:** Anroop B. Nair, Jigar Shah, Bandar E. Al-Dhubiab, Shery Jacob, Snehal S. Patel, Katharigatta N. Venugopala, Mohamed A. Morsy, Sumeet Gupta, Mahesh Attimarad, Nagaraja Sreeharsha, Pottathil Shinu

**Affiliations:** 1 Department of Pharmaceutical Sciences, College of Clinical Pharmacy, King Faisal University, Al-Ahsa 31982, Saudi Arabia; baldhubiab@kfu.edu.sa (B.E.A.-D.); kvenugopala@kfu.edu.sa (K.N.V.); momorsy@kfu.edu.sa (M.A.M.); mattimarad@kfu.edu.sa (M.A.); sharsha@kfu.edu.sa (N.S.); 2 Department of Pharmaceutics, Institute of Pharmacy, Nirma University, Ahmedabad 382481, Gujarat, India; 3 Department of Pharmaceutical Sciences, College of Pharmacy, Gulf Medical University, Ajman 4184, United Arab Emirates; sheryjacob6876@gmail.com; 4 Department of Pharmacology, Institute of Pharmacy, Nirma University, Ahmedabad 382481, Gujarat, India; snehalpharma53@gmail.com; 5 Department of Biotechnology and Food Technology, Durban University of Technology, Durban 4000, Natal, South Africa; 6 Department of Pharmacology, Faculty of Medicine, Minia University, El-Minia 61511, Egypt; 7 Department of Pharmacology, M. M. College of Pharmacy, Maharishi Markandeshwar (Deemed to be University), Mullana 133203, India; sumeetgupta25@gmail.com; 8 Department of Pharmaceutics, Vidya Siri College of Pharmacy, Off Sarjapura Road, Bangalore 560035, India; 9 Department of Biomedical Sciences, College of Clinical Pharmacy, King Faisal University, Al-Ahsa 31982, Saudi Arabia; spottathail@kfu.edu.sa

**Keywords:** clarithromycin, solid lipid nanoparticles, optimization, permeation, pharmacokinetics

## Abstract

Solid lipid nanoparticles (SLNs) are being extensively exploited as topical ocular carrier systems to enhance the bioavailability of drugs. This study investigated the prospects of drug-loaded SLNs to increase the ocular permeation and improve the therapeutic potential of clarithromycin in topical ocular therapy. SLNs were formulated by high-speed stirring and the ultra-sonication method. Solubility studies were carried out to select stearic acid as lipid former, Tween 80 as surfactant, and Transcutol P as cosurfactant. Clarithromycin-loaded SLN were optimized by fractional factorial screening and 3^2^ full factorial designs. Optimized SLNs (CL10) were evaluated for stability, morphology, permeation, irritation, and ocular pharmacokinetics in rabbits. Fractional factorial screening design signifies that the sonication time and amount of lipid affect the SLN formulation. A 3^2^ full factorial design established that both factors had significant influences on particle size, percent entrapment efficiency, and percent drug loading of SLNs. The release profile of SLNs (CL9) showed ~80% drug release in 8 h and followed Weibull model kinetics. Optimized SLNs (CL10) showed significantly higher permeation (30.45 μg/cm^2^/h; *p* < 0.0001) as compared to control (solution). CL10 showed spherical shape and good stability and was found non-irritant for ocular administration. Pharmacokinetics data demonstrated significant improvement of clarithromycin bioavailability (*p* < 0.0001) from CL10, as evidenced by a 150% increase in C_max_ (~1066 ng/mL) and a 2.8-fold improvement in AUC (5736 ng h/mL) (*p* < 0.0001) as compared to control solution (C_max_; 655 ng/mL and AUC; 2067 ng h/mL). In summary, the data observed here demonstrate the potential of developed SLNs to improve the ocular permeation and enhance the therapeutic potential of clarithromycin, and hence could be a viable drug delivery approach to treat endophthalmitis.

## 1. Introduction

Ophthalmic drug delivery continues to pose challenges to formulation scientists due to the complex biochemical, anatomical, and physiological ocular barriers. The biological membranes that protects the anterior and posterior segments of the eye, in addition to the unique structure of the cornea, result in poor ocular bioavailability [1]. Topical drug administration to the anterior segment of the eye causes large pre-corneal clearance because of their high tear turn over (0.5–2.2 μL/min) and rapid blinking rate (6–15 times/min) [2]. Consequently, conventional ophthalmic dosage forms must be applied with frequent instillations to attain and/or control targeted drug levels within the anterior segment of the eye. Delivering the drug molecules to the posterior segment of the eye is challenging primarily due to the long diffusion pathway and the cellular characteristics of the vitreous humor [3]. Therefore, alternative techniques are utilized to transport drugs to the posterior vitreous, the uveal tract, retina, or choroid [4]. Endophthalmitis is a severe intraocular inflammatory infection that affects the vitreous and aqueous fluids within the anterior and posterior region of eye [5]. It is the infection generally caused due to organisms such as bacteria, fungi, or parasites that enter the eye through the blood stream, surgery in the eye or other parts near the eye, or sepsis [6]. A literature review indicated that few drug delivery systems have been developed and their efficacy evaluated in animals. In one attempt, the therapeutic effect demonstrated by an intravitreal drug delivery system of voriconazole was superior to intravitreal injection in rabbits [7]. Chitosan nanoparticles containing daptomycin were developed in ocular treatment of bacterial endophthalmitis. It was reported that these nanoparticles had appropriate characteristics to recommend as a non-invasive method for the ocular delivery of daptomycin to the eye [8].

Clarithromycin is a broad spectrum macrolide antibiotic and shows potential action against various organisms, including *Staphylococcus aureus*, *Streptococcus pneumoniae*, *Legionella pneumophila*, *Moraxella catarrhalis*, *Chlamydia trachomatis*, and *Mycobacterium avium* [9]. Clarithromycin is chemically 6-*O*-methylerythromycin (C_38_H_69_NO_13_), with a molecular weight of 747.95 Dalton, pKa value of 8.99, and melting point ranges between 217 and 220 °C [10]. Different solubility studies demonstrated that clarithromycin is soluble in acetone, slightly soluble in methanol, ethanol, and acetonitrile, and practically insoluble in water (0.33 mg/L). Lipophilicity is a major determining factor in a compound’s absorption, distribution in the body, penetration across vital membranes and biological barriers, metabolism, and excretion. The pharmacokinetics of clarithromycin in adults showed average oral bioavailability of 53%, plasma concentration between 2.41 and 2.85 µg/L after 500 mg dose, and a half-life of ~4 h [10,11]. Pharmacokinetic and pharmacodynamic investigations suggest that clarithromycin exhibits a similar interaction profile as that of erythromycin [12]. Lipid-based drug delivery system have the potential capacity to entrap both lipophobic and lipophilic drugs, enhance the bioavailability of low aqueous soluble drugs, and protect them against degradation. In the last few decades, liquid nanoemulsions have been increasingly used as drug carriers for lipophilic drugs [13]. However, the feasibility of controlled drug release from nanoemulsions is restricted due to the submicron globule size and the fluid state of the carrier. Alternatively, solid matrices of nanostructured lipid carriers (NLCs) and solid lipid nanoparticles (SLNs) help to improve the stability and safety and provide controlled drug release, and they can incorporate both hydrophilic and hydrophobic molecules and can be used in various routes [14]. The basic difference between NLCs and SLNs is the type of lipids used, where liquid lipids are used in NLCs and solid lipids in SLNs [14].

SLNs are nano-sized (10–1000 nm) particles formed when the solid lipids are dispersed in aqueous media containing surfactants as stabilizers [15]. They widely accepted as a prospective delivery system similar to other colloidal carriers, including liposomes and other polymeric nanoparticles [16,17]. Due to the inclusion of physiologically compatible lipids of either natural or synthetic origin, nonirritant and nontoxic properties in SLNs reduce the potential threats of acute or chronic toxicity [15]. Furthermore, advantages such as sustained and controlled drug release, good physical stability, resistance to degradation of lipids, in vivo acceptability, and ability to increase pre-corneal retention time make SLNs a very adaptive carrier for various drug delivery systems [18]. Indeed, formulations of SLNs can be performed in the absence of organic solvents, can be sterilized, and can also use excipients that are “generally recognized as safe” (GRAS), and hence can be easily scaled-up in industry [19]. The incorporation of lipids in the solid state compared to the liquid form is very effective to obtain controlled drug release, since drug mobility is significantly retarded in solid lipid when compared to liquid oil. Extensive research has been carried in the last two decades to tap the potential of SLNs in delivering drugs through various routes, including the ocular route [19,20,21,22]. The literature suggests that SLNs have been widely studied in the treatment of ocular inflammations, infections, glaucoma, cataracts, age related macular degeneration, and as gene therapy carriers [23]. In light of this, the objective of the present study was to design, formulate, and evaluate the potential of drug-loaded SLNs to improve the therapeutic efficacy of clarithromycin in ocular therapy. Fractional factorial design was applied for preliminary screening of various factors utilized in the SLN formulation. A 3^2^ full factorial design was employed to evaluate the influence of independent variables on the dependent variables. Selected SLN were assessed for drug permeation using goat cornea and evaluated in vivo in rabbits.

## 2. Materials and Methods

### 2.1. Materials

Clarithromycin was provided by Century Pharmaceuticals Ltd., Vadodara, India. Stearic acid, glyceryl monostearate, Tween 20, Tween 80, Span 80, sodium lauryl sulphate, polyethylene glycol 400 (PEG 400), and propylene glycol were commercially procured from Central Drug House, New Delhi, India. Compritol 888ATO, Gelucire 43/01, Precirol ATO5, Geleol, and Transcutol P were procured from Gattefosse India, Mumbai, India. Monegyl T18 and Softemul^®^ were purchased from Mohini Organics, Mumbai, India. Stearylamine was obtained from Hi-Media, Mumbai, India.

### 2.2. High Performance Liquid Chromatography (HPLC)

Estimation of clarithromycin was done using a Jasco HPLC system (LC–4000, Easton, MD, USA) containing a degasser unit, pump, an automatic injector, as well as an MD–4010 UV–Visible detector and a Phenomenex C-18 column (150 × 4.6 mm, i.d 5 μm). Chromatographic separation of clarithromycin was performed with the help of a mobile phase consisting of acetonitrile: potassium dihydrogen ortho-phosphate (0.02 mM) 40:60 *v*/*v*, adjusted to pH 5.5 with phosphoric acid [24]. The temperature in the HPLC column was set at 40 °C, and an isocratic elution of clarithromycin was achieved by allowing the solvent to flow at a fixed rate (1 mL/min) and was monitored at 210 nm. Regression analysis indicated good linearity when clarithromycin concentration was 30–400 ng/mL (r^2^ = 0.990).

### 2.3. Solubility of Drug in Lipids and Surfactants

Preliminary screenings were carried out to evaluate the solubility of clarithromycin in various solid lipids and surfactants according to a method described in the literature [25]. Briefly, a weighed quantity (10 mg) of lipid or surfactant was taken in glass vials and melted above its melting point on a water bath (EIE Instruments, Ahmedabad, India). To the melted solution of lipids or surfactant, 10 mg clarithromycin was added and subsequently vortexed. Then, additional increments of lipid or surfactant were added until the drug was completely solubilized or form a homogenous mixture. The lipid in which clarithromycin showed maximum solubility, and the surfactant where it exhibited minimum solubility were selected for the development of an SLN formulation.

### 2.4. Formulation of SLN Using Fractional Factorial Screening Design

SLNs containing clarithromycin, lipid, surfactant, and cosurfactant were formulated by high-speed stirring and the ultra-sonication method described in the literature [26]. High speed mixing generally produces an emulsion, which on cooling forms SLNs. Based on the solubility study of drug in lipid, the amounts of lipid used for the screening design were 150 mg and 200 mg, and the concentrations of surfactants selected were 1 and 3%. Preliminary studies were carried out using different surfactant ratios (surfactant to co-surfactant; 1:9, 3:7, 5:5, 7:3, and 9:1) and evaluated for percent entrapment efficiency and percent drug loading. The selection of the surfactant ratio was based on the target set for percent entrapment efficiency (>85%) and for percent drug loading (>30%).

Screening designs were used to screen the main significant effects from various potential factors that can affect the formulation. Fractional factorial design is one type of screening design. It was applied for screening of all the selected factors utilized for the development of a clarithromycin-loaded SLN formulation. Based on the number of factors, the total number of experimental runs was calculated as sixteen by the equation, 2*^n^*^−1^, where *n* = number of factors. Selected independent variables included for the screening method were process-related factors, such as homogenization speed and sonication time, in addition to formulation variables like amount of lipid, surfactant concentration, and surfactant ratio. The dependent variables investigated were particle size (R_1_), entrapment efficiency (R_2_), and drug loading (R_3_). The coded and actual values of independent variables for fractional factorial screening design are shown in Table 1. 

In brief, an accurately weighed quantity of stearic acid and clarithromycin were solubilized in ethanol by heating on a water bath at 65–70 °C, while surfactant and co-surfactant were dissolved in aqueous phase. SLNs were obtained by slowly adding the molten organic phase drop wise into the aqueous phase under continuous stirring using a homogenizer (Remi Motors, Mumbai, India) set at respective speeds for screening design batches, as shown in Table 2. The precipitated SLN-containing dispersions were subjected to further mixing in cold water using a probe sonicator (Brookfield Engineering Laboratories, MA, USA). The SLNs formed were washed with distilled water and filtered. Table 2 shows the coded and actual values for 16 batches of fractional factorial design.

### 2.5. Evaluation of Screening Design Batches

#### 2.5.1. Particle Size Characterization and Zeta Potential

Particle size analysis, polydispersity index, and zeta potential of the clarithromycin-loaded SLN were determined using a zetasizer (Nanopartica SZ-100, Horiba, Kyoto, Japan). To perform the test, drops (2–3) of test samples placed in the disposable cuvette were exposed to the laser beam. The intensity and physical characteristics of scattered light were measured with the detector, and the particle sizes were subsequently determined [27]. The electrophoretic mobility values were measured after suitable dilution with water at 25 °C.

#### 2.5.2. Percent Drug Entrapment Efficiency and Percent Drug Loading

Formulations were filled in vials and centrifuged at 15,000 rpm using an ultracentrifuge (Remi Motors, Mumbai, India) for 25 min to separate the supernatant and pellets. The pellets were dissolved in methanol, and the drug contents were measured using HPLC. Entrapment efficiency and drug loading were calculated using the formula described in the literature [28].

### 2.6. Optimization Design

A 3^2^ full factorial design was used to assess the influence of independent variables on the dependent variables for the prepared clarithromycin SLNs. Based on the fractional factorial design data, the two factors of amount of lipid (mg) (X1) and sonication time (min) (X2) showed substantial effects on the particle size, drug entrapment efficiency, and drug loading, and hence were applied at 3 levels. In addition, the other three factors, namely homogenization speed, surfactant ratio, and surfactant concentration, were kept constant at 9000 rpm, 5:5, and 3%, respectively, during the experimental run of nine experiments. The dependent variables were Y_1_: particle size; Y_2_: percent entrapment efficiency; and Y_3_: percent drug loading. Table 3 shows coded and actual values of the independent variables of the 3^2^ full factorial design.

The responses Y_1_, Y_2_, and Y_3_ were evaluated using the equation Y = b_0_ + b_1_X_1_ + b_2_X_2_ + b_12_ X_1_X_2_ + b_11_X_11_ + b_22_X_22_. This equation represents the main factors (X_1_ and X_2_), interaction factors (X_1_X_2_), and polynomial factors (X_11_ and X_22_). The b_0_ is the mathematic mean value of total runs, and b_1_ and b_2_ are the projected coefficients for the factors X_1_ and X_2_, respectively. For validation of selected statistical design, polynomial equations were generated using Design Expert software, and one-way analysis of variance (ANOVA) was tested. With the help of response surface plots, the correlation between variables and response parameters was established. The method of preparation was the same as per the screening design batches. Table 4 shows the coded and actual values for 9 batches of the 3^2^ full factorial design.

### 2.7. Evaluation of Design Batches of SLN

#### 2.7.1. Drug Release

A Franz diffusion cell (Logan Instruments Ltd., Somerset, NJ, USA) having an exposed surface area of 0.79 cm^2^, was used to carry out in vitro drug release. In short, a semipermeable membrane (MWCO 12–14 kDa) was held between the donor and receptor compartment. Clarithromycin-loaded SLN dispersion (CL1–CL9, 2.5 mg/mL of drug) or control (drug solution in 10% *v*/*v* span 80) was added in the upper compartment. The acceptor compartment (20 mL) contained simulated tear fluid (pH 7.4) with 1% Tween 80 (to maintain sink conditions) [29] and was stirred at 50 rpm. The temperature of the entire assembly was controlled at 37 ± 0.5 °C by means of a thermostatic water bath. At various time intervals, aliquots of samples (1 mL) were drawn and substituted with the equivalent amount of buffer held at the same temperature. A control experiment was performed at the same experimental conditions using a similar strength of clarithromycin solution. The samples were later diluted with mobile phase and quantified for clarithromycin. The data collected were analyzed to calculate regression coefficient (r^2^) and interpret various mathematical models [30].

#### 2.7.2. Trans-Corneal Permeation

Ex vivo permeation experiments were performed utilizing the vertical Franz diffusion cell using an isolated goat corneal membrane obtained from a local abattoir (Ahmedabad, India) held between the donor and receptor cell [31]. Testing were done using either optimized formulation (CL10) or control (0.25% drug solution equivalent to 2.5 mg of clarithromycin/mL). The receptor compartment was filled with simulated tear fluid (pH 7.4) with 1% Tween 80 (to maintain sink conditions), and temperature was set at 37 ± 0.5 °C. Suitable volumes of the samples were taken at specific time intervals and similar volumes of fluids at the same temperature. The samples withdrawn from the receptor compartment were appropriately diluted and estimated for clarithromycin by HPLC. The flux was determined as described in the literature [32]. 

#### 2.7.3. Transmission Electron Microscopy (TEM)

TEM (Holland Technai 20, Phillips, Amsterdam, The Netherlands) with bright field imaging and magnification technique was employed to examine the physical characteristics of SLN, such as particle size, shape, and surface morphology. A drop of the SLN dispersion was allowed to fix to the surface of a carbon coated copper grid, and the excess of dispersion was then wiped off using the tip of a filter paper. A drop of negative staining solution, 1% (*w*/*v*) phosphotungstic acid, was applied to the affixed SLNs, and extra stain was removed with a tissue paper [33]. The films were formed on grids by air drying at room temperature, and images were captured at 80 kV by TEM.

### 2.8. Ocular Compatibility

The biocompatibility of the optimized formulation (CL10) was performed in six albino rabbits (2–3 kg). The experiments were performed in accordance with the guidelines stated by the Committee for the Purpose of Control and Supervision of Experiments on Animals (approval number IP/PCEU/FAC/21/029, dated; 9 June 2017). Eye irritation score was measured according to the guidelines based on the Draize technique [34]. Single topical instillation of 50 µL of product (CL10) was applied on the left eye of each rabbit, whereas the same volume of physiological saline (reference) was applied on the right eye. The sterile formulation was tested two times a day for 21 days. The cornea, iris, and conjunctiva of rabbits were frequently observed during the study period. Similarly, signs of sensitivity reactions, particularly redness, swelling, cloudiness, edema, hemorrhage, discharge, and blindness, were checked [35].

### 2.9. Pharmacokinetics

The amount of clarithromycin diffused into the aqueous humor of the rabbit eyes after ophthalmic administration was determined to compare the ocular bioavailability between CL10 and control (solution). In vivo pharmacokinetic investigations were performed in New Zealand Albino rabbits (2–3 kg) with two groups (*n* = 6). The experiments were carried out by strictly following the guidelines for animal care at Nirma University (IP/PCEU/FAC/21/029; dated 9 June 2017). Single topical instillation (60 μL of 0.25% *w*/*v* drug) of SLN was dropped in one eye of individual rabbit in the first group, while the second eye remained untreated. A similar strength and volume of control solution was instilled into the second group of rabbits. Both eyelids of all rabbits were lightly closed for 2 min to increase the contact of drug with the corneal membrane. Before aqueous humor withdrawal, individual animals were anaesthetized by intramuscular administration of xylazine and ketamine [36]. The aqueous humor was collected using 29-gauge insulin syringe needle [37], and 20 μL of sample was mixed with acetonitrile and centrifuged, and the organic layer was assessed for clarithromycin content by HPLC.

### 2.10. Stability

An accurately weighed quantity of optimized SLN (CL10) was sealed separately in light resistant glass vials at ambient temperature (25 ± 1 °C) and under refrigerated conditions (4 ± 0.5 °C) up to 3 months [38]. The stability of SLN was examined by assessing the drug content, particle size, and drug release during the storage period.

### 2.11. Statistical Analysis

The software used for Statistical data analysis was GraphPad Prism software (version 6, GraphPad, San Diego, CA, USA). The difference in values at *p* < 0.05 was considered statistically significant.

## 3. Results and Discussion

### 3.1. Solubility

**Figure 1:**
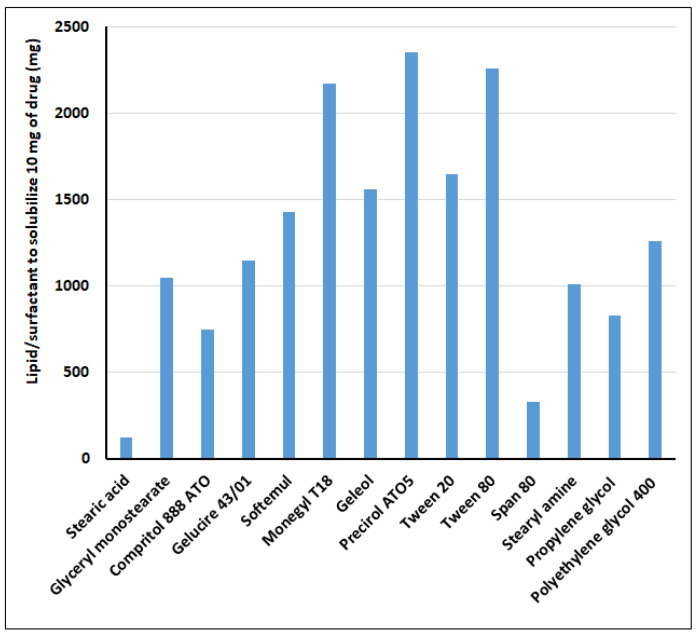
Solubility data showing the amount of lipid/surfactants required to solubilize 10 mg of clarithromycin.

The solubility of drug in lipid is necessary for the preparation of SLNs. The solubility of clarithromycin in solid lipids and surfactants was carried out by a modified method [25], as the normal equilibrium solubility method is not realistic for solid lipids. The lipid in which the drug is most soluble was selected, as low drug solubility in lipid can lead to a decrease in encapsulation efficiency and drug loading [14]. In addition, low drug lipid solubility may prevent binding of drug molecules to the lipid, and the drug may remain as free drug and thereby fail to provide sustained release as desired. From the data shown in Figure 1, it was concluded that the least amount of stearic acid was required to solubilize clarithromycin when compared with other lipids tested. In general, stearic acid has been widely used in ophthalmic drug delivery systems, and in particular in formulating SLNs [39]. Similarly, surfactants are also important components in SLNs, as they play a crucial role in the physicochemical properties, dissolution, permeation, and stability of particles [40]. However, the high solubility of the drug in surfactant may cause leaching of the actives out of the solid lipid particles of the prepared formulations [41]. Therefore, a combination of surfactants was selected in such a manner that the drug would have minimum solubility in the surfactants. The applicability of the surfactant and cosurfactant in preparing single phase micro or nanoemulsions was described elsewhere in the literature [42]. Based on solubility criteria, the non-ionic surfactant Tween 80 was selected as the surfactant and Transcutol P as the cosurfactant for the preparation of various SLN formulations. Tween 80 is often used in various conventional ophthalmic formulations since it does not induce ocular sensitivity reactions [43]. Additionally, due to longer hydrocarbon chain length and larger polar head groups, Tweens may expand the zone of the emulsion region [42]. In addition, Transcutol P can enhance the corneal permeability of the drug through different transport mechanisms [44].

The existence of several colloidal structures such as molecular solubilized emulsifier monomers, mixed micelles, supercooled melts, and drug nanoparticles are important points to consider in SLN formulation. Micelle-forming surfactants are mainly present either on the lipid surface, in micelles or as monomers. The kinetics of drug redistribution between various coexisting colloidal species is very important since the dynamic phenomena are significant for drug stability and drug release [45]. The SLN system can be an efficient carrier system provided that the redistribution of drug between various colloidal forms are prevented. A wide range of drugs with varying lipophilicity has been extensively investigated with respect to their incorporation into SLNs, which signifies the localization of drug within the solid lipid matrix [46]. The log P value of 3.16 for clarithromycin indicates that the drug is mostly located in the lipid matrix. Drug stabilization is a major formulation challenge in colloidal carriers such as SLN due to the enormous surface area and short diffusional pathways. High viscosity provided by lipids actively decreases the diffusion coefficient of the drug inside the carrier system.

### 3.2. Evaluation of Fractional Factorial Screening Batches of SLN

The evaluation of fractional factorial screening batches of prepared SLN is shown in Table 5. The prepared sixteen batches were evaluated for particle size, percent entrapment efficiency, and percent drug loading.

#### 3.2.1. Effect on Particle Size

**Figure 2:**
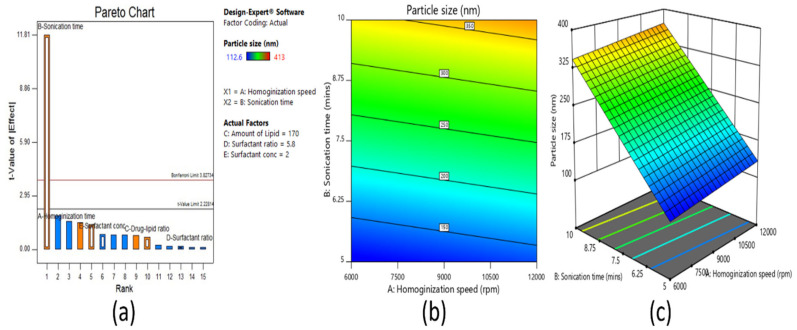
Pareto chart (**a**), contour plot (**b**), and 3D surface plot (**c**) representing the effect on particle size of fractional factorial design.

**Figure 3:**
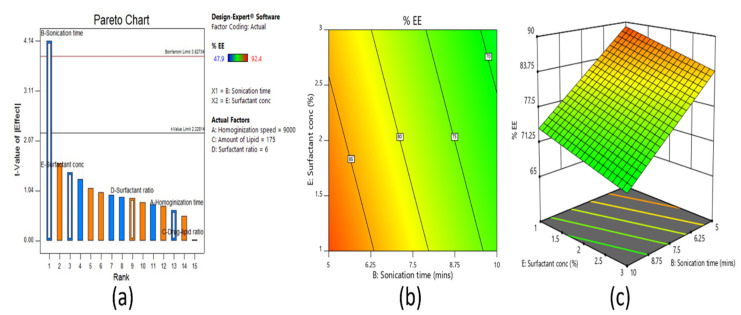
Pareto chart (**a**), contour plot (**b**), and 3D surface plot (**c**) representing the effect on percent entrapment efficiency of fractional factorial design.

The particle size diameter of the SLN dispersions prepared was between 113 nm to 413 nm, as shown in Table 5. From the ANOVA study, it was observed that particle size was significantly (*p* < 0.05) affected by sonication time (min), and it was also confirmed from the Pareto chart (Figure 2a) that t-value of sonication time was above the Bonferroni critical limit, so it had a more significant effect on SLN size. The contour (Figure 2b) and 3D surface plot (Figure 2c) showed that as sonication time decreased from 10 min to 5 min, the size of SLNs also decreased from 413 to 113 nm. This is possible because the sonication can break down the large emulsion drops into tiny particles [25]. On the other hand, the polydispersity index values noticed were in the range of 0.12–0.40 (Table 5).

#### 3.2.2. Effect on Entrapment Efficiency

Entrapment efficiency refers to the quantity of clarithromycin that was entrapped either within the solid matrix or adsorbed on the surface of nanoparticles. It was quantified by analyzing the amount of drug present in the solid pellets as well as in the aqueous phase of the nanoparticle dispersion. It was concluded from the Pareto chart (Figure 3a) that the sonication time had a substantial influence on the entrapment efficiency. The majority of the formulations possessed high entrapment efficiency of >70%, which also revealed the role of surfactant concentration and ratio, amount of lipid, and homogenization speed on drug incorporation. The selection of suitable stabilizing agents, such as surfactants, and their concentration can have a major impact on the entrapment efficiency of SLN dispersions [45]. Surfactants not only present on the lipid surface but also exists as micelles in the aqueous phase. Micelles and mixed micelles are well known to solubilize the drugs and thus act as an alternate drug localization site. In the current study, sonication likely resulted in more interaction between the particles in lipid and aqueous phases due to the ultrasonic shock waves created by the cavitation forces in the liquid, as described in the literature [47]. These intense forces would have probably caused a rapid transfer of clarithromycin to the core of lipid matrix and subsequently increased entrapment percentage at an optimum sonication time. The contour plot presented in Figure 3b and 3D surface plot in the Figure 3c also confirmed that as sonication time increased (from 5 min to 10 min), the percent entrapment efficiency decreased. This reduction in entrapment efficiency could be due to the extension of sonication time, which in turn would have resulted in greater disintegration of agglomerates, which results in leaking of drug particles from lipid vesicles and thereby decreases the percent entrapment efficiency [48]. However, when the surfactant concentration decreased, a minor improvement in the percent entrapment efficiency was noticed. This could be due to the solubilizing property of surfactant [49] and the existence of an optimum surfactant level, which helped the clarithromycin to stay within the lipid particles and improve the entrapment efficiency of the drug [50].

#### 3.2.3. Effect on Drug Loading

**Figure 4:**
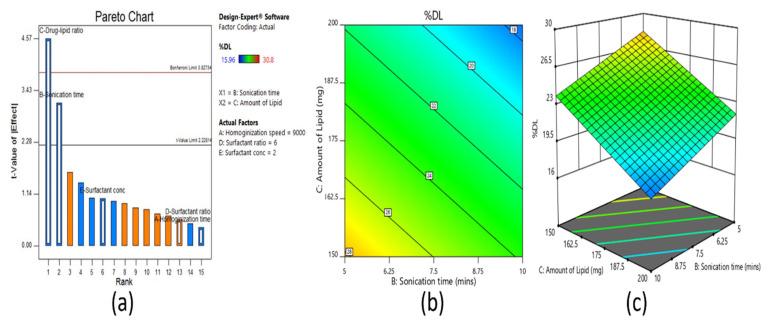
Pareto chart (**a**), contour plot (**b**), and 3D surface plot (**c**) representing the effect on percent drug loading of fractional factorial design.

From the ANOVA study and Pareto chart (Figure 4a), it was observed that the amount of lipid and sonication time had significant effects on percent drug loading, as the t-value was above the limit. The batches with 150 mg of lipid showed good drug entrapment and faster solidification of the nanoparticles. The contour (Figure 4b) and 3D surface response plot (Figure 4c) show that drug loading increased as the sonication time decreased. However, no significant effect in drug loading was noticed with homogenization speed, surfactant concentration, or surfactant ratio.

### 3.3. The 3^2^ Full Factorial Optimization Design

Based on the studies of fractional factorial screening design and data analysis, it was observed that out of five independent variables (including formulation and process variables), two variables (amount of lipid and sonication time) were considered for further optimization. Homogenization speed, surfactant ratio, and surfactant concentration had low or insignificant effects, and hence they were kept constant. A total of nine batches (CL1–CL9) were prepared (Table 6) and were evaluated for particles size, percent entrapment efficiency, and percent drug loading.

### 3.4. Evaluation of Design Batches of SLN

#### 3.4.1. Effect on Particle Size

**Figure 5:**
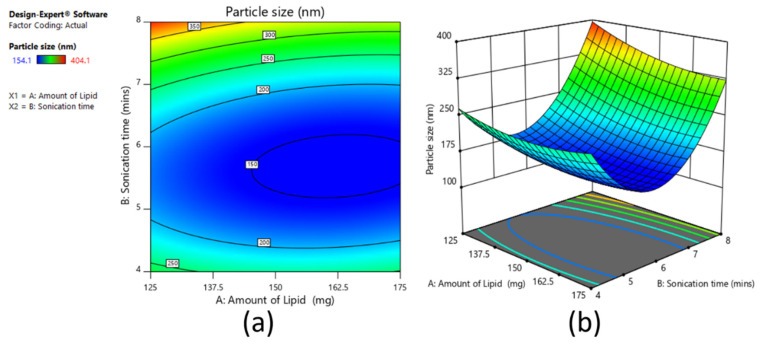
Contour plot (**a**) and 3D surface plot (**b**) representing the effect on particle size for full factorial design batches.

The effect of amount of lipid and sonication time on particle size was studied, and the relevant contour plot and 3D surface response plot are shown in Figure 5a,b, respectively. From the plots, it was observed that as the lipid amount increased from 150 mg to 175 mg, there were increases in particle size (for sonication time 4 and 6 min). This was due to the distribution of sonication energy in the dispersion containing a higher lipid amount dispersion being weaker than in that with the lower lipid amount, which was responsible for more efficient increases in the particle size [25]. However, the same was not observed in the case of 125 mg to 150 mg due to the effect of sonication time. In the case of sonication time, as it increased from 4 to 6 min, particle size decreased, as mentioned earlier, due to more sonication energy reducing the size of the nanoemulsion droplets [25]. However, by further increasing the tie from 6 to 8 min, particle size increased. This was due to more sonication energy, smaller droplet agglomerates, and increases in the size. As per this study, it was observed that a lipid amount of 150 mg and a sonication time of 6 min could be considered as the optimum value. The below polynomial equation (Equation (1)) shows the main, interactive, and polynomial effects of amount of lipid and sonication time on particle size.

Particle size = +150.41 − 21.17 × X_1_ + 50.67 × X_2_ − 14.95 × X_1_ X_2_ + 19.13 × X_11_ + 139.23 × X_22_ (1)

The reduced equation by considering the significant term/s (*p* < 0.05) for particle size can be written as Equation (2):

particle size = +150.41 − 21.17 × X_1_ + 50.67 × X_2_ + 139.23 × X_22_ (2)

On the other hand, the polydispersity index values noticed were in the range of 0.12–0.37 (for sonication times of 4 and 6 min; Table 6), suggesting particles were monodispersed [51], while they were polydispersed when the sonication time was 6 min.

#### 3.4.2. Effect on Entrapment Efficiency

**Figure 6:**
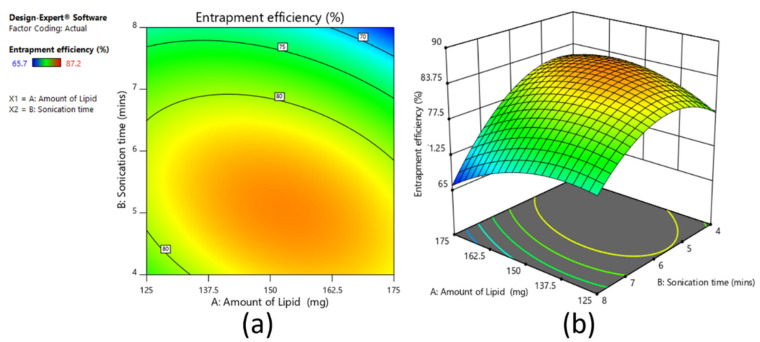
Contour plot (**a**) and 3D surface plot (**b**) representing the effect on percent entrapment efficiency for full factorial design batches.

Figure 6a,b shows the contour plot and 3D surface plot on the effect of the amount of lipid and sonication time on percent entrapment efficiency. It was observed that as sonication time increased, percent entrapment efficiency increased up to 6 min and then decreased from 6 min to 8 min. This was due to higher sonication, which increased the mixture viscosity (with increased lipid concentration) and increased drug solubility within the lipid core and thereby increased the entrapment efficiency [52]. In this study, at lower sonication time (4 min), as the lipid amount increased, entrapment efficiency increased from 79.6% to 81.9%. The reverse condition was observed in cases of higher sonication time (8 min); as the lipid amount increased, the percent entrapment efficiency decreased from 74.9% to 65.7%. However, at medium sonication time (6 min), near to a significant difference was observed in the entrapment efficiency, initially increasing from 76.9% to 87.2% and then decreasing to 79.4%, with respect to the lipid amount (150 mg). The batch showed good entrapment efficiency of about 87.2%. In addition, the polynomial equation confirmed that the effect of sonication time was more significant on percent entrapment efficiency than the amount of lipid.

The polynomial equation (Equation (3)) of percent entrapment efficiency is as follows:

Percent entrapment efficiency = +83.19 − 0.73 × X_1_ − 5.10 × X_2_ − 2.87 × X_1_X_2_ − 3.03 × X_11_ − 5.63 × X_22_ (3)

The reduced equation by considering the significant term (*p* < 0.05) for percent entrapment efficiency can be written as Equation (4):

percent entrapment efficiency = − 5.10 × X_2_ (4)

#### 3.4.3. Effect on Drug Loading

**Figure 7:**
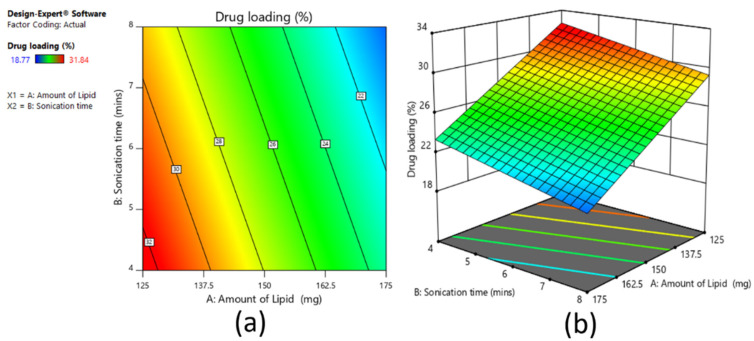
Contour plot (**a**) and 3D surface plot (**b**) representing the effect on percent drug loading for full factorial design batches.

From Figure 7a,b, it was observed that as the amount of lipid increases, the percent drug loading decreases. This is due to the fixed drug amount in the formulation, as the drug concentration remained the same regardless of the increased lipid concentration that caused a decrease in the drug:lipid ratio with increasing lipid concentration [25]. For sonication time, also as time increased, there was a minor decrease in the percent of drug loading. The polynomial equation showing a negative sign for both variables indicates that the variables have inverse relations with percent drug loading. The polynomial equation (Equation (5)) is as follows:

Percent drug loading = +26.32 − 4.62 × X_1_ − 1.63 × X_2_ (5)

The reduced equation is the same, as both factors significantly affect percent drug loading.

In summary, it can be concluded that for particle size, sonication time is the key variable, and with 6 min of sonication time, the targeted particle size can be achieved. In terms of percent entrapment efficiency, again sonication time of 6 min would be highly effective, while with increases in the amount of lipid beyond a certain limit, there would be no further increase in percent entrapment efficiency. At last, considering the percent drug loading, the amount of lipid with a significant effect was 125 mg and 150 mg on targeted drug loading and sonication time at 4 min to 6 min. Thus, an overall conclusion from the design study is that around 150 mg amount of lipid and 6 min of sonication time would give the required particle size, percent entrapment efficiency, and percent drug loading.

### 3.5. In Vitro Drug Release

**Figure 8:**
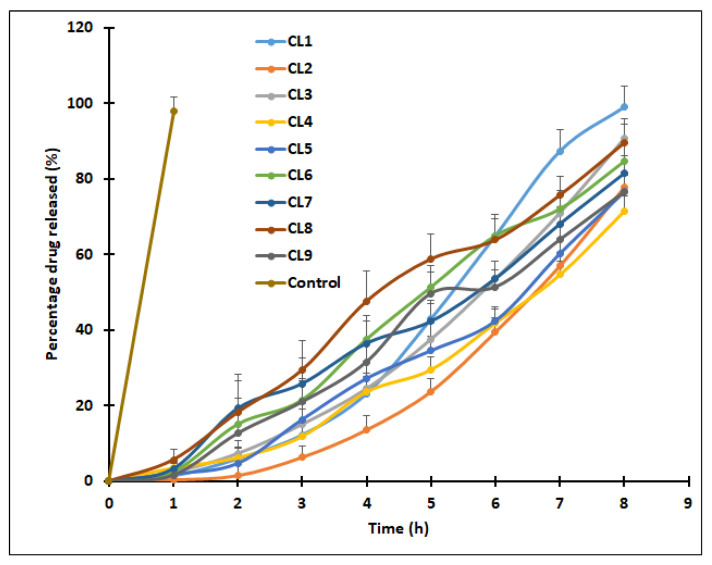
Comparison of percentage of clarithromycin release from solid lipid nanoparticles (CL1–CL9) and drug solution (control). Data represented are mean ± S.D. (*n* = 6).

The release of clarithromycin from SLNs was carried out using a dialysis membrane in Franz diffusion cells. Figure 8 shows the cumulative percentage release of clarithromycin SLNs at specified time intervals from CL1 to CL9. Figure 8 signifies that the drug release profiles of various formulations showed similar patterns of initially slow and then progressively increasing over the time and was higher than 70% in 8 h for all SLNs tested. It was reported that SLN can retard the drug release when the chosen drug has a higher melting point compared to the lipid matrix [46]. It was also disclosed that other factors such as interactions between drug–lipid and surfactant–lipid, as well as solubility of drug in the lipid can play a major role in the drug release from SLNs [53]. Thus, one of the possible explanations for the observed drug release profile could be due to the large differences in the melting point of clarithromycin and stearic acid used. In addition, such release is also possible due to the higher lipid viscosity contributed by the drug–lipid interaction combined with slow diffusion of clarithromycin from the waxy matrix. Amongst all the designed formulations, CL9 had the lowest particle size (154 nm), higher entrapment efficiency (87.2%), and good drug loading (29.1%), which were in well-defined ranges targeted for SLN formulation. The CL9 formulation also exhibited higher drug release (~80% in 8 h). However, the release of clarithromycin in solution (control) was quick, and the whole drug was released in 1 h. The mechanism of clarithromycin release kinetics from CL9 batch was studied using standard mathematical models. Goodness of fit models were selected by evaluating the r^2^ value, sum of squares of residuals, and Fischer ratio in order to avoid errors in the prediction of the release mechanism [29]. The data indicated higher r^2^ value (0.9736), least squares of residuals value (121.54), and Fischer ratio value (17.36) with Weibull model kinetics. Furthermore, the diffusion exponent value (*n* < 0.5) implied the Fickian diffusion mechanism of clarithromycin from CL9.

### 3.6. Design Space

**Figure 9:**
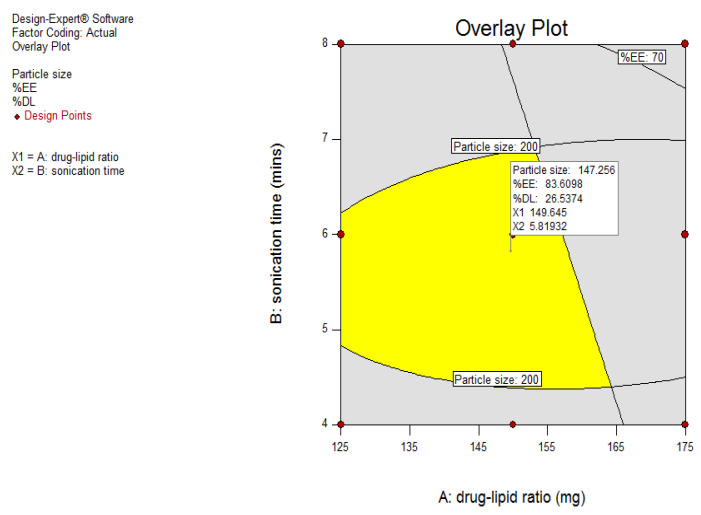
Overlay plot of optimized solid lipid nanoparticle batch using design space.

**Figure 10:**
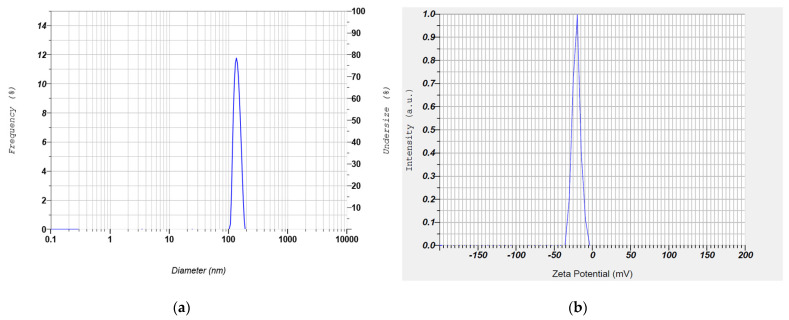
Representative size distribution curve (**a**) and zeta potential distribution (**b**) of optimized clarithromycin solid lipid nanoparticle (CL10).

Since it is difficult to predict the influence of SLN composition and preparation technique on the particle size and percent entrapment efficiency, systematic approaches such as quality by design (QbD) were applied to comprehensively analyze and characterize the design space of the formulation. Using design expert software, design space was obtained by selecting the required range for each dependent variable to obtain the optimized batch. The optimized batch was selected by adding flags to the design space at the design point, which showed that the best response was selected as the optimized batch, and it was further evaluated. Figure 9 shows the overlay plot for the optimized SLN batch (CL10) with low particle size (147 nm), higher entrapment efficiency (83.6%), and good drug loading (26.5%), and its corresponding value of X1 (amount of lipid in mg) was 149.64 and X2 (sonication time in min) was 5.8. Based on these values, the optimized batch was prepared (composition in Table 7). The optimized batch was further evaluated, and the comparison of values (overlay plot-predicted and practical-observed) of dependent variables is shown in Table 8. It was revealed that the predicted values and observed values were similar to each other. The representative size distribution curve and zeta potential distribution of CL10 are depicted in Figure 10.

### 3.7. Trans-Corneal Permeation

**Figure 11:**
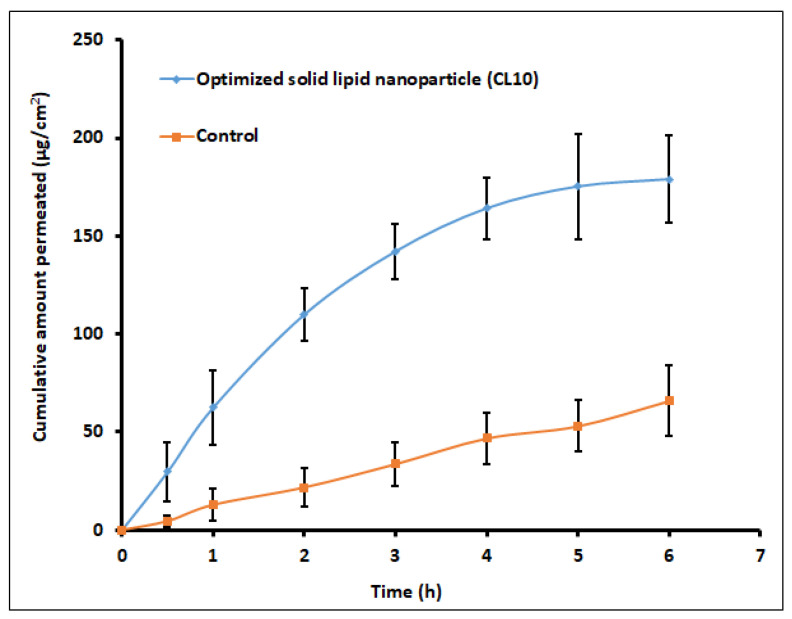
Cumulative amount of clarithromycin permeated through the goat cornea from solid lipid nanoparticles (CL10) and control (solution). Data represented are mean ± S.D. (*n* = 6).

Goat cornea was used for the ex vivo drug permeation investigations for the optimized SLNs (CL10) and control (solution). Various studies have used the goat membrane as a barrier, as this membrane is multi-layered and mimics the human corneal membrane [54]. It is well known that the physico-chemical characteristics of the drug, physiology of the membrane, and the availability of transport routes can affect the permeation of drug molecules into and through the biological membrane [55,56]. The amount of clarithromycin permeated through the cornea membrane from the formulation and control are depicted in Figure 11. Two distinct permeation profiles were noticed, with greater flux (30.45 μg/cm^2^/h; *p* < 0.0001) exhibited by CL10 when compared with control (10.94 μg/cm^2^/h). In the case of CL10, permeation was rapid in the initial 3 h (142.2 ± 14.1 μg/cm^2^), while it was relatively slow and low with the control (33.7 ± 11.1 μg/cm^2^ in 3 h). The greater permeation noticed in the optimized CL10 indicates that the pharmaceutical characteristics of the prepared SLNs (Table 8) are ideal for cornea permeation. Indeed, the higher clarithromycin permeation observed with CL10 suggests that the drug transport could be prominently due to the nanoparticles, as the drug permeation was very low in the control. Moreover, the greater drug concentration above therapeutic levels observed here is also important, as it will avoid microbial resistance. Overall, the observed data here signifies that the SLN could be an ideal carrier for the ocular delivery of clarithromycin.

### 3.8. TEM

**Figure 12:**
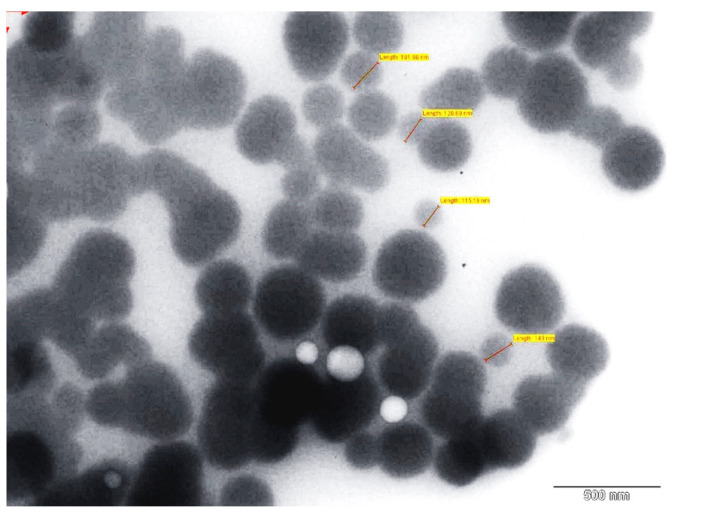
Surface morphology of optimized clarithromycin solid lipid nanoparticles (CL10). Red arrow indicates the diameter of nanoparticle.

A representative TEM micrograph of the optimized SLN (CL10) is shown in Figure 12. The image shows the drug encapsulated in the lipid matrix. It was also observed that the lipid nanoparticles were coarsely spherical in shape and uniformly distributed. The particle size was found to be in the range of 100–200 nm. The particle size range observed in TEM image was in good agreement with the values observed in particle size analysis.

### 3.9. Ocular Compatibility

Eye irritation score from individual rabbits was added to obtain the total irritation score, which was subsequently divided by the total number of eyes used for the ocular irritancy test to obtain the final eye irritation score. The calculated eye irritation score in CL10 was 0.50, while it was 0.17 in the reference. The irritation score observed in CL10 was relatively low and could be classified as practically non-irritant [57]. Further, instillation of CL10 did not cause redness, swelling, or excessive lachrymation in the eye. Overall, the data signified that CL10 is safe and non-irritant for ocular administration.

### 3.10. Pharmacokinetics

**Figure 13:**
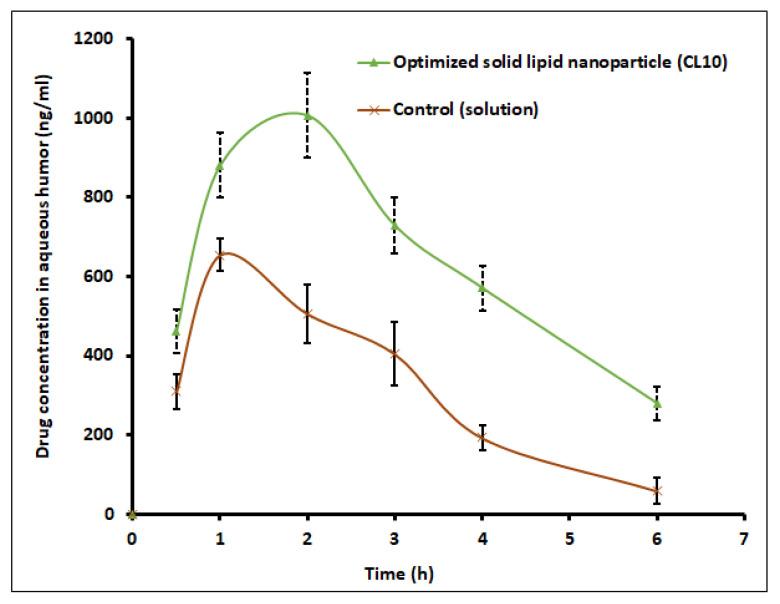
Ocular pharmacokinetics profile of clarithromycin after topical application of solid lipid nanoparticle (CL10) and control (solution) in rabbits. Data represented are mean ± S.D. (*n* = 6).

Ocular bioavailability is calculated as the amount of clarithromycin in the aqueous humor of rabbit eyes of first (CL10) and second groups (control) after single dose administration. The efficacy of topical antibiotics relies on the potential of the formulation to permeate across the corneal tissues and deliver an effective antimicrobial level in the target area. Various pharmacokinetic parameters including t_max_, C_max_, and AUC were computed from the graph plotted between concentrations (ng/mL) in aqueous humor and time (h) by non-compartment model analysis. Data in Figure 13 signify that the drug aqueous humor level in CL10 and control were significantly different throughout the study period in the current experimental conditions. A certain amount of drug was detected in 0.5 h in both cases. The amount of clarithromycin permeated in control experiments could be related to the drug’s lipophilic nature and high partition coefficient. At 1 h, the clarithromycin level increased (881.08 ± 80.96 ng/mL and 654.99 ± 39.53 ng/mL in CL10 and control, respectively), though it was statistically different (*p* < 0.0001). At 2 h, absorption of clarithromycin in CL10 was extended, and the drug level reached ~1000 ng/mL, while the clarithromycin level declined sharply in the control (~500 ng/mL). These data signify that ophthalmic drops were retained in the ocular cavity for a short time because of extensive pre-corneal drug loss through nasolacrimal discharge and tear turnover. Further, the t_max_ value for CL10 was 2 h, while it was 1 h in the control. On the other hand, CL10 showed a 150% increase in C_max_ (~1066 ng/mL) and a 2.8-fold improvement in AUC (5736 ng h/mL) (*p* < 0.0001) as compared to the control solution (C_max_; 655 ng/mL and AUC; 2067 ng h/mL). Thus, it can be concluded from the available data that intraocular permeation of clarithromycin was significantly improved by the optimized SLNs. This observation is also in agreement with ex vivo permeation data, wherein the flux was considerably higher in CL10 (Figure 11). The ocular pharmacokinetics data observed with CL10 were also significantly higher than the values reported in the literature tested with same concentration of clarithromycin eye drops [58]. Therefore, ocular residence time of CL10 proved extended duration of action in comparison to control. The average drug concentration noticed in Figure 13 in the aqueous humor was more than the minimum effective concentration of clarithromycin in various bacterial endophthalmitis [59].

### 3.11. Stability

The optimized clarithromycin SLN (CL10) showed no significant variation in the drug content, particle size, or drug release after 3 months in both storage conditions (4 °C and 25 °C), as noticed in Table 9. Therefore, the stability data indicate good physical stability of the prepared SLNs during their storage at 4 °C and 25 °C for 3 months.

## 4. Conclusions

The solubility of clarithromycin in various lipid and surfactants was studied, and based on solubility criteria, stearic acid (lipid), Tween 80 (nonionic surfactant), and Transcutol P (cosurfactant) were selected for the preparation of SLNs. Initial fractional factorial design suggests that the sonication time and amount of lipid significantly influence the SLN formulation. Further, 3^2^ full factorial design data also confirms that both the factors significantly affect the particle size, drug entrapment efficiency, and drug loading. The drug content and entrapment efficiency of SLNs were enhanced by increasing the concentration of lipid since higher hydrophobicity of the longer chain of stearic acid resulted in a high-loaded clarithromycin. Ex vivo permeation and in vivo pharmacokinetics data signify greater efficacy by the optimized clarithromycin-loaded SLN (CL10). Indeed, the clarithromycin observed in the aqueous humor was significantly higher than the minimum effective concentration of clarithromycin in bacterial endophthalmitis. Further, the topical ocular therapy of clarithromycin could be more advantageous than its oral counterpart because topical administration could minimize the side effects as well as diminish the possibility of producing resistant strains of bacteria. Being a noninvasive approach, topical ocular therapy is more preferred and possesses higher patient compliance in the treatment of various diseases in the anterior segment. Thus, the developed SLN of clarithromycin-loaded nanoparticles has greater potential and can be a promising alternative to conventional therapy for the effective management of bacterial endophthalmitis. Further studies need to be carried out to understand the interaction of SLNs with the biological environment for successful commercialization and regulatory approval.

## Figures and Tables

**Table 1:** Coded and actual values of independent variables for fractional factorial design.

Independent Variable	Levels	
	+1	−1
A: Homogenization speed (rpm)	12,000	6000
B: Sonication time (min)	10	5
C: Amount of lipid (mg)	200	150
D: Surfactant ratio	3:7	5:5
E: Surfactant concentration (%)	3%	1%

**Table 2:** Coded and actual values of fractional factorial design batches.

Batch	Coded Value					Actual Values				
	A	B	C	D	E	A	B	C	D	E
1	1	−1	1	−1	1	12,000	5	200	5:5	3
2	−1	1	1	−1	1	6000	10	200	5:5	3
3	1	1	1	1	1	12,000	10	200	3:7	3
4	−1	1	−1	−1	−1	6000	10	150	5:5	1
5	−1	−1	1	1	1	6000	5	200	3:7	3
6	1	−1	−1	−1	−1	12,000	5	150	5:5	1
7	−1	1	1	1	−1	6000	10	200	3:7	1
8	1	−1	−1	1	1	12,000	5	150	3:7	3
9	1	1	−1	−1	1	12,000	10	150	5:5	3
10	−1	−1	−1	1	−1	6000	5	150	3:7	1
11	−1	−1	−1	−1	1	6000	5	150	5:5	3
12	1	−1	1	1	−1	12,000	5	200	3:7	1
13	−1	−1	1	−1	−1	6000	5	200	5:5	1
14	1	1	1	−1	−1	12,000	10	200	5:5	1
15	−1	1	−1	1	1	6000	10	150	3:7	3
16	1	1	−1	1	−1	12,000	10	150	3:7	1

**Table 3:** Coded and actual values of independent variables of 3^2^ full factorial design.

Independent Variable	Levels		
	+1	0	−1
A: Amount of lipid (mg)	175	150	125
B: Sonication time (min)	8	6	4

**Table 4:** Coded and actual values for 9 batches of the 3^2^ full factorial design.

Batch No.	Coded Value		Actual Value	
	X1	X2		
1	0	+1	150	8
2	−1	+1	125	8
3	+1	+1	175	8
4	+1	0	175	6
5	0	−1	150	4
6	−1	0	125	6
	−1	−1	125	4
8	+1	−1	175	4
9	0	0	150	6

**Table 5:** Evaluation of fractional factorial design batches.

Batch No.	Independent Variables with Actual Values					Dependent Variables			
	Homogenization Speed (RPM)	Sonication Time (Min)	Amount of Lipid (mg)	Surfactant Ratio	Surfactant Concentration (%)	Particle Size (nm)	Polydispersity Index	Entrapment Efficiency (%)	Drug Loading (%)
F1	12,000	5	200	5:5	3	115	0.15	84.9	21.2
F2	6000	10	200	5:5	3	357	0.32	69.9	17.5
F3	12,000	10	200	3:7	3	334	0.40	72.4	18.1
F4	6000	10	150	5:5	1	276	0.26	79.1	26.4
F5	6000	5	200	3:7	3	113	0.12	85.8	21.5
F6	12,000	5	150	5:5	1	124	0.18	92.4	30.8
F7	6000	10	200	3:7	1	369	0.31	72.5	18.1
F8	12,000	5	150	3:7	3	113	0.16	87.9	29.3
F9	12,000	10	150	5:5	3	413	0.29	49.9	15.9
F10	6000	5	150	3:7	1	134	0.18	87.3	26.9
F11	6000	5	150	5:5	3	119	0.14	83.7	27.9
F12	12,000	5	200	3:7	1	130	0.14	85.7	21.4
F13	6000	5	200	5:5	1	124	0.12	84.5	21.1
F14	12,000	10	200	5:5	1	397	0.29	75.2	18.9
F15	6000	10	150	3:7	3	284	0.22	77.1	25.7
F16	12,000	10	150	3:7	1	397	0.26	74.9	24.9

**Table 6:** Evaluation of 3^2^ full factorial design batches.

Batch No.	Coded Value		Particle Size (nm)	Polydispersity Index	Entrapment Efficiency (%)	Drug Loading (%)	Zeta Potential (mV)
	Amount of Lipid (mg)	Sonication Time (Min)					
CL1	150	8	339	0.32	70.7	23.6	−21.3
CL2	125	8	404	0.37	74.9	30.0	−16.7
CL3	175	8	316	0.33	65.7	18.8	−21.9
CL4	175	6	163	0.14	79.4	22.7	−29.8
CL5	150	4	236	0.26	80.4	26.9	−18.8
CL6	125	6	173	0.20	76.9	30.8	−25.4
CL7	125	4	274	0.24	79.6	31.8	−21.6
CL8	175	4	245	0.27	81.9	23.4	−23.4
CL9	150	6	154	0.12	87.2	29.1	−18.5

**Table 7:** Composition of optimized solid lipid nanoparticle batch (CL10) based on design space.

Ingredients	Quantity
Stearic acid	149.64 mg
Tween 80	0.3 mL
Transcutol P	0.3 mL
Water	20 mL
Ethanol	5 mL
Homogenization speed	9000 rpm
Sonication time	5.8 min

**Table 8:** Evaluation of optimized solid lipid nanoparticle batch (CL10).

Parameter	Predicted Value	Observed Value *
Entrapment efficiency (%)	83.6	81.3 ± 4.6
Drug loading (%)	26.5	27.1 ± 3.9
Particle size (nm)	147	157 ± 42.4
Polydispersity index	0.12	0.13 ± 0.02
Zeta potential (mV)	−18.4	−17.2 ± 3.1
In vitro drug release (%)	87	89.4 ± 4.5

* All values are expressed as mean ± S.D.; *n* = 6.

**Table 9:** Stability data of optimized solid lipid nanoparticle batch (CL10) after 3 months.

Parameter	Refrigerated Temperature	Room Temperature
Drug content (%)	97.6 ± 4.9	95.8 ± 4.6
Particle size (nm)	165 ± 54.6	172 ± 61.5
In vitro drug release (%)	88.8 ± 7.1	86.3 ± 5.0

All values are expressed as mean ± S.D.; *n* = 6.

## Data Availability

The data presented in this study are contained within the article.

## References

[1] Shah J., Nair A.B., Jacob S., Patel R.K., Shah H., Shehata T.M., Morsy M.A. (2019). Nanoemulsion Based Vehicle for Effective Ocular Delivery of Moxifloxacin Using Experimental Design and Pharmacokinetic Study in Rabbits. Pharmaceutics.

[2] Lin S., Ge C., Wang D., Xie Q., Wu B., Wang J., Nan K., Zheng Q., Chen W. (2019). Overcoming the Anatomical and Physiological Barriers in Topical Eye Surface Medication Using a Peptide-Decorated Polymeric Micelle. ACS Appl. Mater. Interfaces.

[3] Varela-Fernández R., Díaz-Tomé V., Luaces-Rodríguez A., Conde-Penedo A., García-Otero X., Luzardo-Álvarez A., Fernández-Ferreiro A., Otero-Espinar F.J. (2020). Drug Delivery to the Posterior Segment of the Eye: Biopharmaceutic and Pharmacokinetic Considerations. Pharmaceutics.

[4] Shah J.N., Shah H.J., Groshev A., Hirani A.A., Pathak Y.V., Sutariya V. (2014). Nanoparticulate transscleral ocular drug delivery. J. Biomol. Res. Ther..

[5] Durand M.L. (2013). Endophthalmitis. Clin. Microbiol. Infect..

[6] Kernt M., Kampik A. (2010). Endophthalmitis: Pathogenesis, clinical presentation, management, and perspectives. Clin. Ophthalmol. Auckl. N.Z..

[7] Yang L., Dong X., Wu X., Xie L., Min X. (2011). Intravitreally implantable voriconazole delivery system for experimental fungal endophthalmitis. Retin. Phila. Pa..

[8] Silva N.C., Silva S., Sarmento B., Pintado M. (2015). Chitosan nanoparticles for daptomycin delivery in ocular treatment of bacterial endophthalmitis. Drug Deliv..

[9] Gutiérrez-Castrellón P., Mayorga-Buitron J.L., Bosch-Canto V., Solomon-Santibañez G., de Colsa-Ranero A. (2012). Efficacy and safety of clarithromycin in pediatric patients with upper respiratory infections: A systematic review with meta-analysis. Rev. Invest. Clin..

[10] (2008). Clarithromycin. Tuberculosis.

[11] Rodvold K.A. (1999). Clinical pharmacokinetics of clarithromycin. Clin. Pharmacokinet..

[12] Pai M.P., Graci D.M., Amsden G.W. (2000). Macrolide drug interactions: An update. Ann. Pharmacother..

[13] Ali A., Ansari V.A., Ahmad U., Akhtar J., Jahan A. (2017). Nanoemulsion: An Advanced Vehicle For Efficient Drug Delivery. Drug Res..

[14] Duong V.A., Nguyen T.T., Maeng H.J. (2020). Preparation of Solid Lipid Nanoparticles and Nanostructured Lipid Carriers for Drug Delivery and the Effects of Preparation Parameters of Solvent Injection Method. Mol. Basel Switz..

[15] Borges A., Freitas V., Mateus N., Fernandes I., Oliveira J. (2020). Solid Lipid Nanoparticles as Carriers of Natural Phenolic Compounds. Antioxid. Basel Switz..

[16] Rajpoot K. (2019). Solid Lipid Nanoparticles: A Promising Nanomaterial in Drug Delivery. Curr. Pharm. Des..

[17] Kamboj S., Bala S., Nair A.B. (2010). Solid Lipid Nanoparticles: An Effective Lipid Based Technology for Poorly Water Soluble Drugs. Int. J. Pharm. Sci. Rev. Res..

[18] Wissing S.A., Kayser O., Müller R.H. (2004). Solid lipid nanoparticles for parenteral drug delivery. Adv. Drug Deliv. Rev..

[19] Weber S., Zimmer A., Pardeike J. (2014). Solid Lipid Nanoparticles (SLN) and Nanostructured Lipid Carriers (NLC) for pulmonary application: A review of the state of the art. Eur. J. Pharm. Biopharm..

[20] Paliwal R., Paliwal S.R., Kenwat R., Kurmi B.D., Sahu M.K. (2020). Solid lipid nanoparticles: A review on recent perspectives and patents. Expert Opin. Ther. Pat..

[21] Garcês A., Amaral M.H., Sousa Lobo J.M., Silva A.C. (2018). Formulations based on solid lipid nanoparticles (SLN) and nanostructured lipid carriers (NLC) for cutaneous use: A review. Eur. J. Pharm. Sci. Off. J. Eur. Fed. Pharm. Sci..

[22] Salah E., Abouelfetouh M.M., Pan Y., Chen D., Xie S. (2020). Solid lipid nanoparticles for enhanced oral absorption: A review. Colloids Surf. B Biointerfaces.

[23] Sánchez-López E., Espina M., Doktorovova S., Souto E.B., García M.L. (2017). Lipid nanoparticles (SLN, NLC): Overcoming the anatomical and physiological barriers of the eye—Part II—Ocular drug-loaded lipid nanoparticles. Eur. J. Pharm. Biopharm..

[24] Lu Y., Zhang Y., Yang Z., Tang X. (2009). Formulation of an intravenous emulsion loaded with a clarithromycin-phospholipid complex and its pharmacokinetics in rats. Int. J. Pharm..

[25] Das S., Ng W.K., Kanaujia P., Kim S., Tan R.B. (2011). Formulation design, preparation and physicochemical characterizations of solid lipid nanoparticles containing a hydrophobic drug: Effects of process variables. Colloids Surf. B Biointerfaces.

[26] Severino P., Santana M.H.A., Souto E.B. (2012). Optimizing SLN and NLC by 22 full factorial design: Effect of homogenization technique. Mater. Sci. Eng. C.

[27] Harsha S.N., Aldhubiab B.E., Nair A.B., Alhaider I.A., Attimarad M., Venugopala K.N., Srinivasan S., Gangadhar N., Asif A.H. (2015). Nanoparticle formulation by Büchi B-90 Nano Spray Dryer for oral mucoadhesion. Drug Des. Dev. Ther..

[28] Nair A.B., Al-Dhubiab B.E., Shah J., Attimarad M., Harsha S. (2017). Poly (lactic acid-co-glycolic acid) Nanospheres improved the oral delivery of candesartan cilexetil. Indian J. Pharm. Educ. Res.

[29] Öztürk A.A., Aygül A., Şenel B. (2019). Influence of glyceryl behenate, tripalmitin and stearic acid on the properties of clarithromycin incorporated solid lipid nanoparticles (SLNs): Formulation, characterization, antibacterial activity and cytotoxicity. J. Drug Deliv. Sci. Technol..

[30] Nair A., Gupta R., Vasanti S. (2007). In vitro controlled release of alfuzosin hydrochloride using HPMC-based matrix tablets and its comparison with marketed product. Pharm. Dev. Technol..

[31] Nair A.B., Shah J., Jacob S., Al-Dhubiab B.E., Sreeharsha N., Morsy M.A., Gupta S., Attimarad M., Shinu P., Venugopala K.N. (2021). Experimental design, formulation and in vivo evaluation of a novel topical in situ gel system to treat ocular infections. PLoS ONE.

[32] Nair A.B., Jacob S., Al-Dhubiab B.E., Alhumam R.N. (2018). Influence of skin permeation enhancers on the transdermal delivery of palonosetron: An in vitro evaluation. J. Appl. Biomed..

[33] Shah J., Nair A.B., Shah H., Jacob S., Shehata T.M., Morsy M.A. (2020). Enhancement in antinociceptive and anti-inflammatory effects of tramadol by transdermal proniosome gel. Asian J. Pharm. Sci..

[34] Wilhelmus K.R. (2001). The Draize eye test. Surv. Ophthalmol..

[35] Wilson S.L., Ahearne M., Hopkinson A. (2015). An overview of current techniques for ocular toxicity testing. Toxicology.

[36] Nair A., Morsy M.A., Jacob S. (2018). Dose translation between laboratory animals and human in preclinical and clinical phases of drug development. Drug Dev. Res..

[37] Singh M., Guzman-Aranguez A., Hussain A., Srinivas C.S., Kaur I.P. (2019). Solid lipid nanoparticles for ocular delivery of isoniazid: Evaluation, proof of concept and in vivo safety & kinetics. Nanomed. Lond. Engl..

[38] Nair A.B., Sreeharsha N., Al-Dhubiab B.E., Hiremath J.G., Shinu P., Attimarad M., Venugopala K.N., Mutahar M. (2019). HPMC- and PLGA-Based Nanoparticles for the Mucoadhesive Delivery of Sitagliptin: Optimization and In Vivo Evaluation in Rats. Mater. Basel Switz..

[39] Dubald M., Bourgeois S., Andrieu V., Fessi H. (2018). Ophthalmic Drug Delivery Systems for Antibiotherapy—A Review. Pharmaceutics.

[40] Botto C., Mauro N., Amore E., Martorana E., Giammona G., Bondì M.L. (2017). Surfactant effect on the physicochemical characteristics of cationic solid lipid nanoparticles. Int. J. Pharm..

[41] Parhi R., Suresh P. (2010). Production of solid lipid nanoparticles-drug loading and release mechanism. J. Chem. Pharm. Res..

[42] Pavoni L., Perinelli D.R., Bonacucina G., Cespi M., Palmieri G.F. (2020). An Overview of Micro- and Nanoemulsions as Vehicles for Essential Oils: Formulation, Preparation and Stability. Nanomater. Basel Switz..

[43] Jiao J. (2008). Polyoxyethylated nonionic surfactants and their applications in topical ocular drug delivery. Adv. Drug Deliv. Rev..

[44] Moiseev R.V., Morrison P.W.J., Steele F., Khutoryanskiy V.V. (2019). Penetration Enhancers in Ocular Drug Delivery. Pharmaceutics.

[45] Mehnert W., Mäder K. (2001). Solid lipid nanoparticles: Production, characterization and applications. Adv. Drug Deliv. Rev..

[46] zur Mühlen A., Schwarz C., Mehnert W. (1998). Solid lipid nanoparticles (SLN) for controlled drug delivery—Drug release and release mechanism. Eur. J. Pharm. Biopharm. Off. J. Arb. Pharm. Verfahr..

[47] Leong T.S.H., Martin G.J.O., Ashokkumar M. (2017). Ultrasonic encapsulation—A review. Ultrason. Sonochem..

[48] Pradhan S., Hedberg J., Blomberg E., Wold S., Wallinder I.O. (2016). Effect of sonication on particle dispersion, administered dose and metal release of non-functionalized, non-inert metal nanoparticles. J. Nanopart. Res..

[49] Sharma M., Gupta N., Gupta S. (2016). Implications of designing clarithromycin loaded solid lipid nanoparticles on their pharmacokinetics, antibacterial activity and safety. RSC Adv..

[50] Baig M.S., Ahad A., Aslam M., Imam S.S., Aqil M., Ali A. (2016). Application of Box-Behnken design for preparation of levofloxacin-loaded stearic acid solid lipid nanoparticles for ocular delivery: Optimization, in vitro release, ocular tolerance, and antibacterial activity. Int. J. Biol. Macromol..

[51] Shah H., Nair A.B., Shah J., Jacob S., Bharadia P., Haroun M. (2021). Proniosomal vesicles as an effective strategy to optimize naproxen transdermal delivery. J. Drug Deliv. Sci. Technol..

[52] Khames A., Khaleel M.A., El-Badawy M.F., El-Nezhawy A.O.H. (2019). Natamycin solid lipid nanoparticles—Sustained ocular delivery system of higher corneal penetration against deep fungal keratitis: Preparation and optimization. Int. J. Nanomed..

[53] Zur Mühlen A., Mehnert W. Drug incorporation and delivery of prednisolone loaded solid lipid nanoparticles. Proceedings of the 1st World Meeting APGI/APV.

[54] Ranch K.M., Maulvi F.A., Naik M.J., Koli A.R., Parikh R.K., Shah D.O. (2019). Optimization of a novel in situ gel for sustained ocular drug delivery using Box-Behnken design: In vitro, ex vivo, in vivo and human studies. Int. J. Pharm..

[55] Nair A.B., Chakraborty B., Murthy S.N. (2010). Effect of polyethylene glycols on the trans-ungual delivery of terbinafine. Curr. Drug Deliv..

[56] Nair A.B., Singh K., Al-Dhubiab B.E., Attimarad M., Harsha S., Alhaider I.A. (2013). Skin uptake and clearance of ciclopirox following topical application. Biopharm. Drug Dispos..

[57] Wolska E., Sznitowska M., Chorążewicz J., Szerkus O., Radwańska A., Markuszewski M.J., Kaliszan R., Raczyńska K. (2018). Ocular irritation and cyclosporine A distribution in the eye tissues after administration of Solid Lipid Microparticles in the rabbit model. Eur. J. Pharm. Sci..

[58] Zhang J., Wang L., Zhou J., Zhang L., Xia H., Zhou T., Zhang H. (2014). Ocular penetration and pharmacokinetics of topical clarithromycin eye drops to rabbits. J. Ocul. Pharmacol. Ther. Off. J. Assoc. Ocul. Pharmacol. Ther..

[59] Brockhaus L., Goldblum D., Eggenschwiler L., Zimmerli S., Marzolini C. (2019). Revisiting systemic treatment of bacterial endophthalmitis: A review of intravitreal penetration of systemic antibiotics. Clin. Microbiol. Infect..

